# Regulation of epigenetic modifications in the head and neck tumour microenvironment

**DOI:** 10.3389/fimmu.2022.1050982

**Published:** 2022-10-28

**Authors:** Huimin Liu, Dongxu Wang, Zhijing Yang, Shuangji Li, Han Wu, Jingcheng Xiang, Shaoning Kan, Ming Hao, Weiwei Liu

**Affiliations:** ^1^Department of Oral and Maxillofacial Surgery, Hospital of Stomatology, Jilin University, Changchun, China; ^2^Jilin Provincial Key Laboratory of Tooth Development and Bone Remodeling, Hospital of Stomatology, Jilin University, Changchun, China; ^3^Laboratory Animal Center, College of Animal Science, Jilin University, Changchun, China

**Keywords:** DNA methylation, histone modification, RNA modifications, ncRNAs, head and neck tumours, tumour microenvironment

## Abstract

Head and neck tumours are common malignancies that are associated with high mortality. The low rate of early diagnosis and the high rates of local recurrence and distant metastasis are the main reasons for treatment failure. Recent studies have established that the tumour microenvironment (TME) can affect the proliferation and metastasis of head and neck tumours *via* several mechanisms, including altered expressions of certain genes and cytokines. Increasing evidence has shown that epigenetic modifications, such as DNA methylation, histone modification, RNA modification, and non-coding RNAs, can regulate the head and neck TME and thereby influence tumour development. Epigenetic modifications can regulate the expression of different genes and subsequently alter the TME to affect the progression of head and neck tumours. In addition, the cell components in the TME are regulated by epigenetic modifications, which, in turn, affect the behaviour of head and neck tumour cells. In this review, we have discussed the functions of epigenetic modifications in the head and neck TME. We have further examined the roles of such modifications in the malignancy and metastasis of head and neck tumours.

## Introduction

Globally, head and neck tumours are the most common malignant tumours (1). More than 430,000 people die from the disease annually (1), and 90% of the cases involve head and neck squamous cell carcinoma (HNSCC) (2). It is considered the dominant phenotype and is associated with cervical lymph node metastasis and the progression of malignancy (3). Surgical resection is the mainstay of HNSCC treatment; however, it is often unsatisfactory owing to the uncertainty of tumour boundaries and the potential for aggressive lymphatic metastasis (4). Many therapies are available for head and neck tumour microenvironment (TME), including immunotherapy and anti-angiogenic therapy (5, 6). Immunological monotherapy and combination therapy have been shown to prolong the survival of patients (7), and anti-angiogenic therapy is an attractive option for overcoming hypoxia and radiation in head and neck tumours (6). Both immunotherapy and anti-angiogenic therapy are closely linked to alterations in the TME (8, 9). Therefore, it is vital to explore the impact of TME on head and neck tumours.

The TME includes both cellular and non-cellular components, such as stromal cells, immune cells, and chemokines (10). The processes of proliferation, apoptosis resistance, invasion, migration, and immune evasion of cancer cells are considered to be related to TME (11-15). The acidic and hypoxic TME creates a unique growth environment for cancer cells, which enables them to resist the immune response (16, 17). The TME can influence tumour progression *via* multiple mechanisms, such as Notch and STAT3 signalling pathways (18, 19). Furthermore, m^6^A modifications are involved in the regulation of TME. For example, the m^6^A demethylase ALKBH5 can regulate the tumour immune microenvironment (TIME) of HNSCC *via* the RIG-I/IFNα signalling pathway (20), which suggests that epigenetic modifications are involved in the regulation of the TME in head and neck tumours. Therefore, the characteristics of the TME should be explored, and the mechanisms regulating epigenetic modifications of the TME in HNSCC must be clarified.

Epigenetic alterations lead to abnormal gene expression in the cells in the TME and are linked to the development of cancer (21). Recent studies have shown that TME is regulated by epigenetic modifications (22). In this review, we have summarised the interactions between epigenetic modifications and the TME as well as their effects on the fate of HNSCC. These insights may help in identifying new potential targets for the effective treatment of head and neck tumours.

## The TME of head and neck tumours

**Figure 1:**
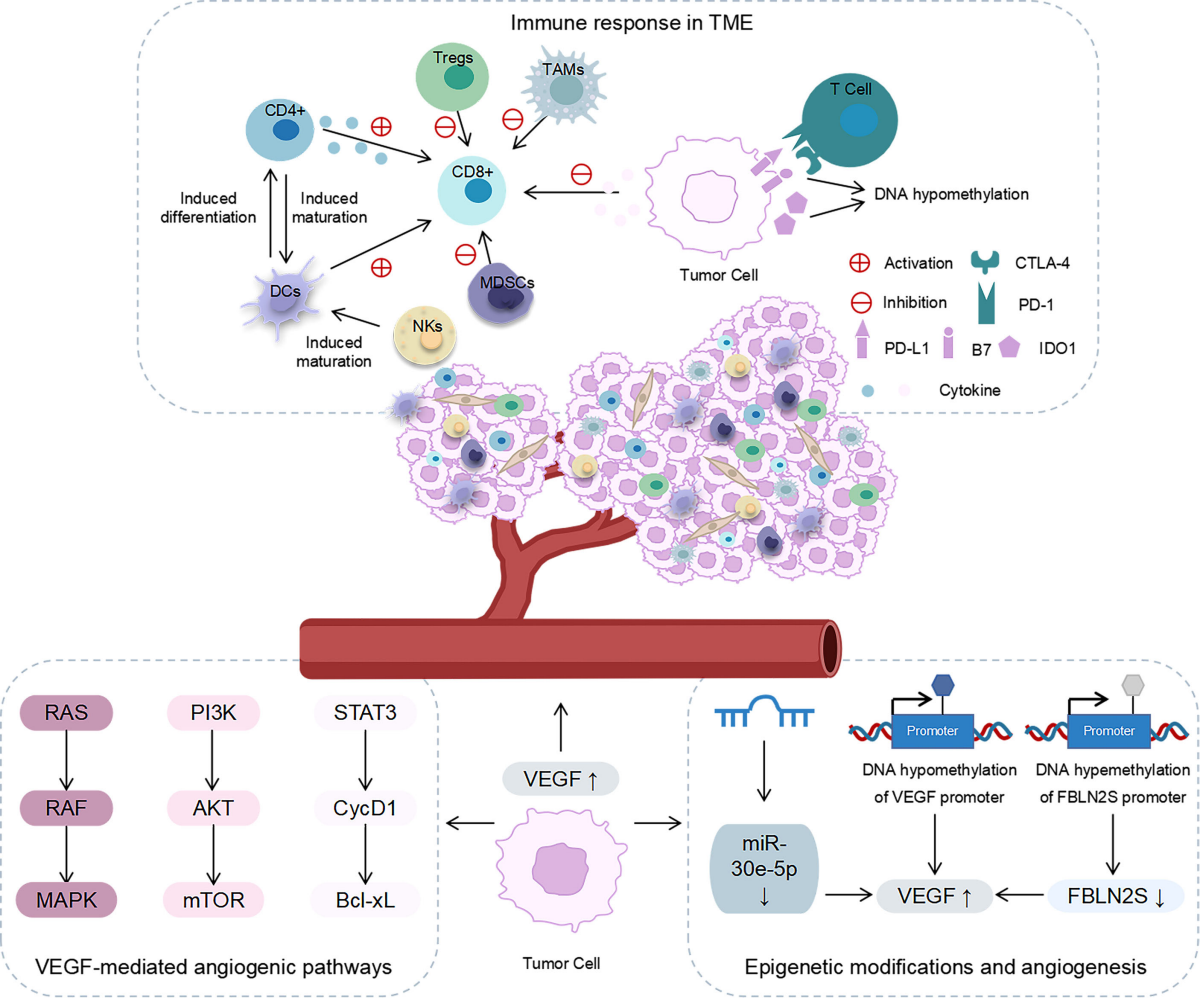
The role of angiogenesis and immune response in the TME. Immune response in the TME: NKs and CD4^+^ induced DC maturation; DCs promoted the activation of CD8^+^T cells and induced CD4^+^T cells to differentiate into antigen-specific effector T cells; CD4^+^ secreted cytokines to activate CD8^+^T cells; MDSCs, Tregs and TAMs inhibited the tumour-killing effect of CD8^+^T cells; cancer cells released cytokines to inhibit T-cell proliferation and the effector function; PD-1/PD-L1 interaction inhibited the function of effector T cells; CTLA-4 binds to B7 ligands to inhibit T cell activation; IDO1 inhibited the function of T cells; the expression levels of CTLA-4 and IDO1 were regulated by DNA methylation. Angiogenesis in the TME: VEGF affected angiogenesis through RAS/RAF/MAPK, PI3K/AKT/mTOR, and STAT3/cycD1/Bcl-xL signalling pathways. Epigenetic modification affected the expression of VEGF and the angiogenesis in the TME.

The microenvironment of head and neck cancer is a complex system that is composed of non-tumour cells, an extracellular matrix, and a vascular system (23). The TME is characterized by hypoxia, high angiogenic factor content, and immunosuppression and is involved in tumour growth, metastasis, and invasion (6, 24, 25). In addition to conventional approaches, such as surgical treatment, radiotherapy, and chemotherapy, anti-angiogenic and immunosuppressive therapies are novel directions for treating these tumours. Therefore, we focussed on the role of the TME in angiogenesis and immune responses (**Figure 1**).

### The role of angiogenesis in the TME of head and neck tumours

The rapid proliferation of cells in malignant tumours requires a supply of oxygen and nutrients *via* the bloodstream (26). Angiogenesis is a key factor that affects the progression of head and neck cancers (27, 28). Microvessel density (MVD) is an indirect marker of tumour angiogenesis and is associated with poor prognosis in HNSCC (29). Disease-free survival and overall survival are significantly reduced in patients with high CD105^+^ MVD (29). A study has reported that increased MVD in head and neck tumours promotes the proliferation of cancer cells and leads to poor tissue differentiation and lymph node metastasis (30). Therefore, the mechanism of angiogenesis in head and neck cancer needs to be explored for developing targeted angiogenic therapy.

The downstream signalling pathway of angiogenesis is mainly mediated by vascular endothelial growth factor (VEGF) (31). It affects angiogenesis chiefly *via* three signalling pathways, namely, RAS/RAF/MAPK, PI3K/AKT/mTOR, and STAT3/cycD1/Bcl-xL (6). In head and neck tumours, VEGF-C has been observed to promote cell growth and migration (32). Recent studies have revealed that epigenetic modifications can directly or indirectly regulate the expression of VEGF and, thus, play a role in the angiogenesis in head and neck tumours. For instance, sevoflurane can reduce the angiogenic ability of tongue squamous cell carcinoma by enhancing DNA methylation in the VEGF promoter region (33). In nasopharyngeal carcinoma, high DNA methylation of the FBLN2S promoter inhibits the expression of FBLN2S, while overexpression of FBLN2S downregulates the expression of angiogenesis-related factors such as VEGF165 and VEGF189 and inhibits angiogenesis (34). Moreover, non-coding RNAs (ncRNAs) play a vital role in angiogenesis. A study has suggested that upregulating the expression of miR-30e-5p can inhibit mRNA expression of VEGF and inhibit angiogenesis in HNSCC *in vivo*, thereby reducing tumour invasion and metastasis (35). Based on these findings, it is clear that epigenetic modifications are instrumental in the angiogenesis in head and neck tumours and may provide a new target for anti-angiogenic treatment.

### The role of immune responses in the TME of head and neck tumours

The role of the immune system in tumour development is widely accepted, and therapeutic modalities targeting tumour immunity have been frequently reported (36). To facilitate the development of immunotherapy for head and neck cancer, the components involved in immunotherapy, such as immune cells and immune checkpoints, deserve to be investigated.

#### The role of immune cells in the TME of head and neck tumours

The TME of solid tumours contains a variety of immune cells, such as CD8^+^ T cells, CD4^+^ T cells, natural killer cells (NKs), and dendritic cells (DCs), which act as anti-tumour agents. The TME also contains myeloid suppressor cells (MDSCs), regulatory T cells (Tregs), and tumour-associated macrophages (TAMs), which act as immunosuppressive agents (37, 38). These immune cells, along with cytokines, constitute TIME. DCs promote the activation of CD8^+^ T cells and induce CD4^+^ T cells to differentiate into antigen-specific effector T cells (39). CD4^+^ T cells and NKs induce the maturation of DCs and contribute to the activation of CD8^+^ T cells (40, 41). Furthermore, CD4^+^ T cells exert their tumour-killing effect by secreting cytokines that activate CD8^+^ T cells (42). The TME of solid tumours presents a highly immunosuppressed state. MDSCs, Tregs, and TAMs inhibit the tumour killing effect of CD8^+^ T cells and effectively achieve immunosuppression (43-45). Moreover, cancer-associated fibroblasts (CAFs), a major cellular component of the TME matrix, are involved in the immune processes of head and neck tumours. For example, in nasopharyngeal carcinoma, there is a significant correlation between the densities of CAFs and M2 TAMs (46). Furthermore, tumour cells can release immunosuppressive mediators to exert their immunosuppressive effects. Head and neck tumour cells can avoid detection by T cells and NKs by secreting various cytokines, including TGF-β, IL-6, and IL-10, which can inhibit T-cell proliferation and effector function (47–49). Another study has reported that exosomes from peripheral blood of head and neck patients containing cyclooxygenase 2, TGF-β, programmed death 1 (PD-1), and cytotoxic T lymphocyte antigen 4 (CTLA-4). These exosomes promote CD8^+^ T-cell apoptosis, inhibit CD4^+^ T-cell proliferation, upregulate Tregs and impair the function of NKs (50).

#### The role of immune checkpoints in the TME of head and neck tumours

Some immune checkpoints play a role in the immune escape of head and neck tumours, including PD-1/PD-L1, CTLA-4, and indoleamine 2,3-dioxygenase 1 (IDO1). In HNSCC, the expressions of PD1 and PD-L1 are upregulated, which represents one of the key immune checkpoints in HNSCC (51–53). PD-1 is mainly expressed on the surface of T cells and interacts with PD-L1 and PD-L2 ligands (54). The interaction of PD-1 and PD-L1 can inhibit the function of effector T cells and promote immunological tolerance (55). Furthermore, CTLA-4 can bind to B7 ligands on cancer cells, which results in the inhibition of T-cell activation and the promotion of HNSCC immune escape (56). IDO1 is a new immunosuppressive locus whose increased expression can inhibit the function of anti-tumour T cells (57). Recent studies have stated that epigenetic modifications are also involved in the immunomodulatory process in head and neck cancers. Investigations have suggested that CTLA-4 and IDO1 expression levels in head and neck tumours are epigenetically regulated *via* DNA methylation (57, 58). This finding implies that epigenetic modifications are involved in the TME of head and neck tumours. Therefore, the role of epigenetics in the TME of head and neck cancers must be clarified.

## Epigenetic modifications of the TME in head and neck tumours

### DNA methylation of the TME in head and neck tumours

Multiple studies have suggested that DNA methylation is involved in genetic alterations in tumour cells and in activities in the TME (**Figure S1**) (59, 60). A recent study showed that inhibiting the expression of DNA methyltransferase 1 can reduce MDSCs and increase tumour-infiltrating T cells to prevent tumour growth in the TME of oral squamous cell carcinoma (OSCC) (61). Furthermore, altered DNA methylation of certain genes can affect immune cells and, therefore, the TME. In HNSCC, squalene cyclooxygenase is demethylated and overexpressed, which inhibits the activation of CD8^+^ T cells and leads to immunity evasion (62). Another study observed that the TME of patients with ornithine aminotransferase hypomethylation had a higher degree of immune cell infiltration and ornithine aminotransferase hypermethylation exhibited higher radiosensitivity (63).

One study indicated that immune checkpoints in the TME of head and neck tumours were regulated by DNA methylation (58). According to a recent study on oral cancer, the expressions of the immune checkpoint CTLA-4 and its function-related molecules CD28, ICOS, CD80, and CD86 were regulated by DNA methylation (58). Expression of CTLA4 is negatively correlated with DNA methylation, whereas mRNA levels of CD28, ICOS, CD80, and CD86 are positively correlated (58). This evidence suggests that DNA methylation directly or indirectly influences the regulation of the TME in head and neck tumours.

### Histone acetylation of the TME in head and neck tumours

Histones comprise the core histones H2A, H2B, H3, and H4 and the connective histone H1 (64). Acetylation of histone H4-Lys16 and trimethylation deletion of H4-Lys20 are common markers of human cancer (65). Many studies have shown that histone deacetylase inhibitors (HDACis) can reshape the TME and enhance the ability of the immune system to kill tumour cells (66–69). A recent study opined that head and neck tumours sustain low levels of histone acetylation, which may be the reason for the accumulation and maintenance of cancer stem cells (70). The above evidence implies that histone acetylation has an essential role in the TME of head and neck tumours.

### RNA modification of the TME in head and neck tumours

#### m^1^A modification

m^1^A modification is closely related to the TIME in several cancers (71, 72). m^1^A modification has been documented to affect the TME in HNSCC and thereby influence its prognosis. A recent study has shown that the m^1^A gene mutation may be associated with the TME of OSCC and that it could potentially predict its prognosis (73). Researchers have analysed the methylation pattern of m^1^A, which revealed that the expressions of all m^1^A regulatory factors were significantly upregulated in 502 patients with OSCC compared with the normal control group. This upregulation was closely linked to poor prognosis in the patients (73). Moreover, m^1^A modification was shown to be negatively correlated with immune checkpoints, angiogenesis, and CD8^+^ T cells in the TME (73). A study has reported that this modification can affect the TME in head and neck tumours by influencing the long non-coding RNAs (lncRNAs). Furthermore, m^1^A-related lncRNA has been observed to be closely related to the prognosis, TME, and tumour mutation burden in HNSCC (74). In summary, m^1^A modification exerts immunomodulatory effects in the TME of HNSCC.

#### m^5^C modification

m^5^C modification is a key player in several biological and pathological processes, such as cell proliferation and differentiation, tumorigenesis, malignant tumour progression, and tumour immunity (75, 76). Moreover, m^5^C regulatory factors can regulate the TIME (77). A recent study has stated that the activities of most immune cells in the TME are significantly reduced in patients with a high expression of m^5^C regulators (78). This observation implies a correlation between the TME and m^5^C in patients with OSCC (78). Furthermore, m^5^C can influence the TME *via* lncRNAs. Another study has suggested the presence of a close relationship between m^5^C-related lncRNA and the HNSCC TME (74). In HNSCC, m^5^C regulators inhibit immune cell activity, which means that m^5^C can regulate the TME and influence its fate.

#### m^6^A modification

m^6^A modification is the most common mRNA modification in eukaryotes (79). This modification is involved in the regulation of RNA stability, localisation, output, splicing, and translation and is closely linked to several cellular activities (80). Abnormal m^6^A modification is associated with a TME phenotype of non-inflammation and immunorejection (81). For instance, low expression of the m^6^A writer METTL3 promotes the production of IL-8. Tumour-associated neutrophils are recruited to the TME for immunosuppression, which promotes the progression of papillary thyroid carcinoma (82). Moreover, m^6^A eraser acts in the TME, with a recent study reporting that the expression of ALKBH5 is upregulated in HNSCC (20). A subsequent study has shown that ALKBH5 downregulates the expression of RIG-I and reduces the secretion of interferon-α in the TME *via* the IKK-α/Tbk1/IRF3 pathway. Ultimately, the infiltration of immune killer cells is inhibited, and immune escape is promoted (20). Furthermore, m^6^A readers YTHDF1 and IGF2BP2 have been found to be significantly correlated with different immunological states in HNSCC. These readers may regulate the TME by blocking the expression of specific genes related to antigen recognition, signal transduction, and effector T-cell proliferation and activation (83). YTHDC2 is associated with the degree of immune infiltration of B cells, CD8^+^ T cells, CD4^+^ T cells, neutrophils, and DCs in HNSCC (84). High expression of YTHDC2 is accompanied by high immune infiltration (84). Moreover, m^6^A is associated with immune checkpoints in the TME. A report has suggested that the upregulation of PD-L1 expression is associated with m^6^A methylation (85). Also, m^6^A methylation and the PI3K/AKT/mTOR signalling pathway may be involved in the regulation of the HNSCC immune microenvironment (85). These reports suggest that m^6^A modifications are primarily associated with immune regulation of the TME in head and neck tumours.

### Non-coding RNAs

ncRNAs, including circular RNAs (circRNAs), microRNAs (miRNAs), and lncRNAs, interact with the components of the TME, thereby affecting tumorigenesis and progression (**Table S1**) (86–88). These ncRNAs, which are enriched in exosomes of CAFs, are transmitted to the cancer cells to regulate their biological characteristics. The decreased expressions of miR-34a-5p and miR-3188 in the CAF exosomes augment the metastatic potential of head and neck tumours (89, 90). miR-382-5p transported by CAF exosomes can promote migration and invasion in OSCC (91). In addition, miR-196a transported by CAF exosomes can augment cisplatin resistance in head and neck tumours by targeting CDKN1B and ING5 (92). Moreover, miRNAs from cancer cells can influence the TME. miR-192/215 transported by exosomes in head and neck tumour cells could promote remodelling of the hypoxic TME (93). Another study has reported that deletion of the p53 gene results in the reduced expression of miR-34a, which promotes adrenergic trans-differentiation of tumour-associated sensory nerves and head and neck tumour progression (94). Furthermore, the downregulation of miR-34a promotes immune escape in head and neck tumours *via* upregulation of MET expression (95). Low levels of miR-9 can promote tumour growth by upregulating MDK expression and regulating the PDK/AKT signalling pathway to enhance angiogenesis in the TME of nasopharyngeal carcinoma (96). Therefore, miRNA plays a key role in the TME of head and neck tumours.

The role of lncRNAs in the microenvironment of head and neck cancer is yet to be elucidated. Like miRNAs, lncRNAs from the TME can influence the fate of the tumour. The lncRNA *H19* is upregulated in CAF and participates in the glycolytic pathway of CAF *via* the miR-675-5p/PFKFB3 axis, which promotes the progression of oral cancer (97). The lncRNA *FLJ22447* is significantly upregulated in CAFs and promotes the transformation of CAF into OSCC by upregulating IL-33 (87). Furthermore, lncRNA transported by tumour cell exosomes can act on components in the TME to regulate tumour progression. Oral leukoplasia (OL) is a precancerous state of OSCC (98). A study has shown that the lncRNA *IFITM4P* can induce PD-L1 expression, thereby activating the immunosuppressive process and immune escape of OL cells in the cytoplasm (98). The expression of the lncRNA *DCST1-AS1* in OSCC is significantly increased, and the polarization of M2 macrophages is promoted by regulation of the NF-κB pathway to enhance tumour progression (99). These results suggest that ncRNA can regulate the TME of head and neck tumours and might serve as a potential therapeutic target.

## Discussion

Head and neck tumours are associated with a poor prognosis because of their high degree of malignancy and the high rate of lymph node metastasis (3). At present, the major treatment for these tumours is surgical resection (4). However, owing to their location, surgical resection of head and neck tumours often results in physiological disorders that affect swallowing, mastication, or pronunciation. Moreover, it can exert a negative influence on the facial appearance and mental health of patients (100, 101). Therefore, therapies that are more efficient and less damaging should be immediately developed. Several studies have shown that the TME can regulate tumorigenesis, growth, and metastasis of head and neck tumours (102–105). As mentioned above, angiogenesis promotes the invasion and metastasis of head and neck tumour, and increased immunosuppression promotes the immune escape of tumour cells (8, 32). Therefore, TME is extremely important for the pathological progression of head and neck tumours. Anti-angiogenic drugs and immunotherapies that target the TME of head and neck tumours are promising alternative therapies (103, 106). However, these treatment modalities require further research to refine them. Therefore, elucidating the role of the TME in head and neck tumours is the need of the hour.

**Figure 2:**
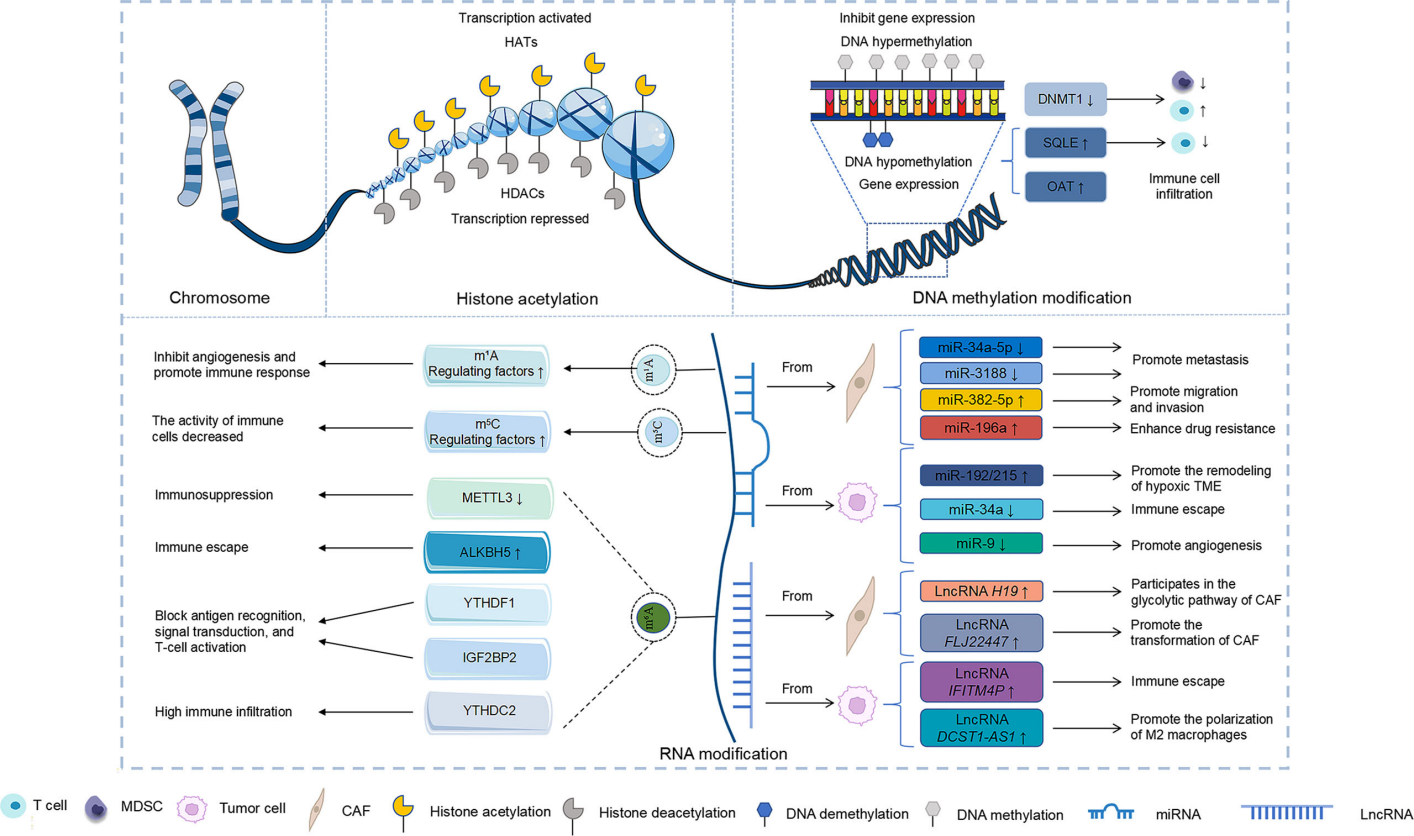
Effect of epigenetic modifications on the TME of head and neck tumours. DNA methylation, RNA modification, and ncRNA modification resulted in different gene expression changes that affected the TME of head and neck tumours.

Epigenetic mechanisms are involved in a variety of pathological processes and play an essential role in tumour progression (21). These alterations can influence the TME of head and neck tumours and regulate cancer progression (**Figure 2**). In this regard, DNA methylation can affect tumour progression by regulating immune infiltration and immune checkpoints in the TME of head and neck tumours (58, 61, 62). Furthermore, histone acetylation can weaken the immune-killing capability of the TME in head and neck tumours and promote their growth (70). RNA modification predominantly regulates the level of angiogenesis, immune activity, and immune infiltration of immune cells in the TME of head and neck tumours and participates in tumour progression (20, 73, 78, 82–85). ncRNA secreted by certain cells in the TME of head and neck tumours can influence the behaviour of cancer cells, including invasion, metastasis, and drug resistance (89, 97). Furthermore, ncRNA in tumour cells can participate in the immune regulation of the TME in head and neck tumours and promote their progression (95, 96, 99). Succinctly, epigenetic modification holds promising potential in the regulation of the TME in head and neck tumours and is expected to provide targets for their treatment. Key proteins in epigenetic modifications can affect angiogenesis, immune responses in TME and can further affect tumour growth (**Table S2**) (61, 73, 78, 82). Numerous drugs targeting different epigenetic modifications have been used to treat head and neck tumours. The major DNA methylation drugs include zebularine, 5-azacytidine, 5-aza-2’-deoxycytidine, aloe-emodin, and procaine (107–109). Trichostatin A, suberoylanilide hydroxamic acid, M344 (an analogue of hydroxamic acid), and cyclic tetrapeptide are potent HDAC inhibitors that have been reported to promote radiosensitivity in head and neck tumours (110). Additionally, the HDAC inhibitor LBH589 promotes p21 expression and induces cell death (111). Therefore, the development of drugs targeting epigenetic modifications in the TME may offer new prospects for the treatment of head and neck tumours.

## Conclusion

The data summarized herein establishes that the TME can affect the malignant development of head and neck tumours, including their growth, metastasis, and drug resistance. Epigenetic modifications are involved in these processes. It is essential to gain more knowledge about the molecular mechanisms involved in epigenetic modifications of TME in head and neck tumours. Therefore, the development of new drugs effectively targeting epigenetic modifications can be envisaged in the near future. In general, clarifying the role of epigenetic modifications in the TME can provide a novel therapeutic target for head and neck tumours.

## Author contributions

HL and DW wrote the manuscript. HL, WL, ZY, SL, HW, JX, SK, and MH collected the references and prepared figures. All authors reviewed the manuscript. All authors listed have made a substantial, direct, and intellectual contribution to the work and approved it for publication.

## Funding

This work was supported by the Fundamental Research Funds for the Jilin Province Department of Finance (grant no. jcsz2021893-13), the Changchun Scientific and Technological Development Program (grant no. 21ZY26) and the Jilin Province Scientific and Technological Development Program (grant no. 20200801077GH, 20210204013YY, 20200504005YY, 20220505033ZP).

## Conflict of interest

The authors declare that the research was conducted in the absence of any commercial or financial relationships that could be construed as a potential conflict of interest.

## Publisher’s note

All claims expressed in this article are solely those of the authors and do not necessarily represent those of their affiliated organizations, or those of the publisher, the editors and the reviewers. Any product that may be evaluated in this article, or claim that may be made by its manufacturer, is not guaranteed or endorsed by the publisher.

## Supplementary material

Supplementary Figure 1 The role of DNA methylation in TME of head and neck tumours. **(A)** DNA hypomethylation of VEGF and DNA hypermethylation of FBLN2S promote angiogenesis. **(B)** Inhibition of DNMT1 expression decreased the percentage of MDSCs and increased CD8^+^T cell infiltration. **(C)** Hypomethylation at CpG S_Shore site of SQLE promotes immune escape. **(D)** OAT hypomethylation promotes immune cell infiltration, and hypermethylation tumour is more sensitive to radiotherapy.
