# Supplementary figures and images for: Regulation of epigenetic modifications in the head and neck tumour microenvironment
Source: Front Immunol. 2022 Oct 28;13:1050982. doi: 10.3389/fimmu.2022.1050982 (PMC9667738; doi:10.3389/fimmu.2022.1050982)

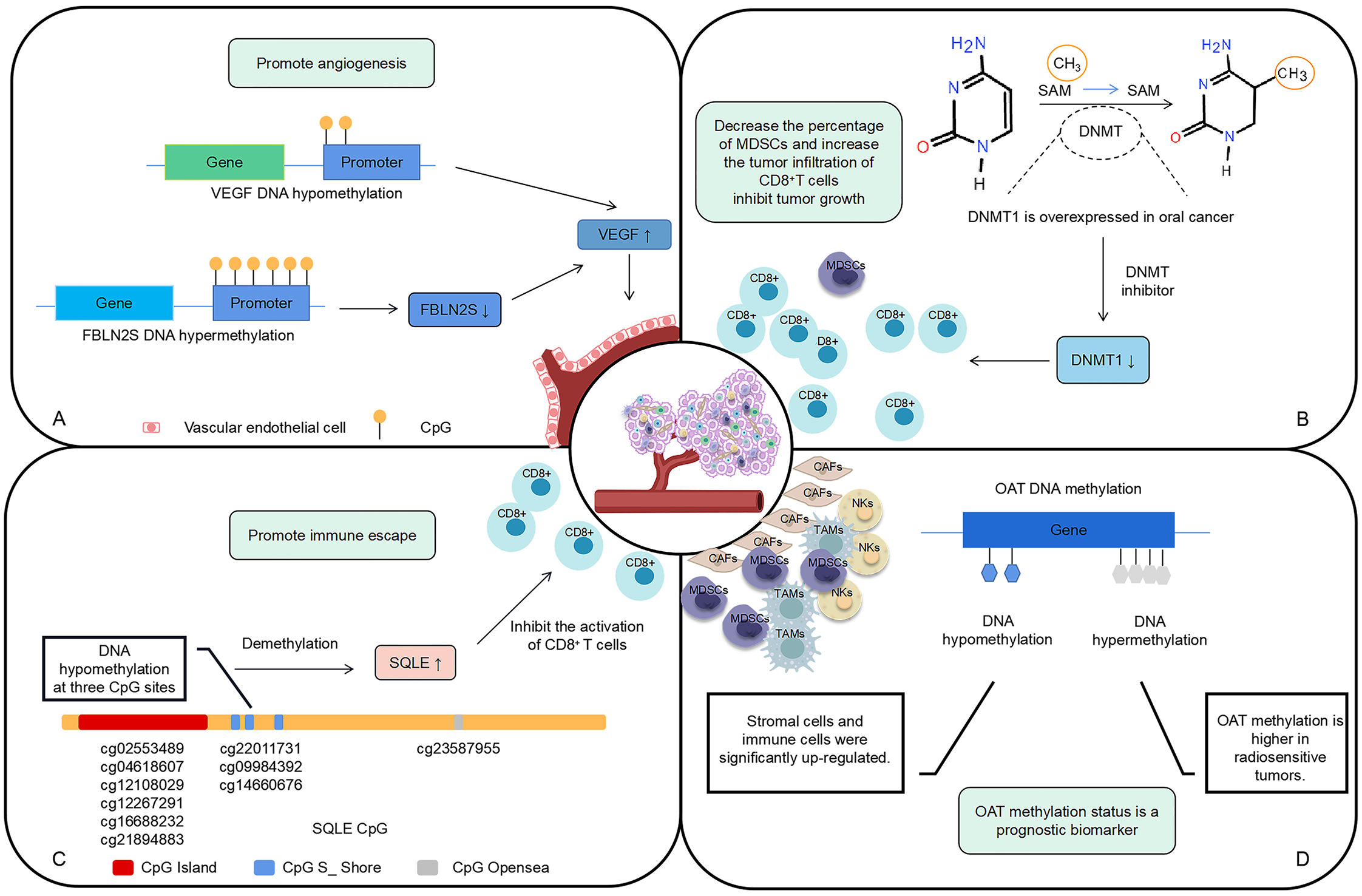

Supplement: Supplementary Figure 1 — The role of DNA methylation in TME of head and neck tumours. (A) DNA hypomethylation of VEGF and DNA hypermethylation of FBLN2S promote angiogenesis. (B) Inhibition of DNMT1 expression decreased the percentage of MDSCs and increased CD8+T cell infiltration. (C) Hypomethylation at CpG S_Shore site of SQLE promotes immune escape. (D) OAT hypomethylation promotes immune cell infiltration, and hypermethylation tumour is more sensitive to radiotherapy. [file Image_1.tif]
