# Supplementary material for: Regulation of epigenetic modifications in the head and neck tumour microenvironment
Source: Front Immunol. 2022 Oct 28;13:1050982. doi: 10.3389/fimmu.2022.1050982 (PMC9667738; doi:10.3389/fimmu.2022.1050982)
Supplement: Supplementary file 2 [file Table_1.docx]

Table S1. The role of ncRNA of the TME in head and neck tumours

| ncRNA | Origin | Expression level | | Role | Ref |  |
| --- | --- | --- | --- | --- | --- | --- |
| miR-34a-5p | CAFs | Down | Increases the metastatic potential | | 1 |  |
| miR-3188 | CAFs | Down | Increases the metastatic potential | | 2 |  |
| miR-382-5p | CAFs | Up | Promote migration and invasion | | 3 |  |
| miR-196a | CAFs | Up | Promote cisplatin resistance | | 4 |  |
| miR-192/215 | Oral cancer cell | Up | Promote remodelling of a hypoxic TME | | 5 |  |
| miR-34a | Oral cancer cell | Down | Promote adrenergic trans-differentiation of tumour-associated sensory nerves and head and neck tumour progression | | 6 |  |
|  | Oral cancer cell | Down | Promote immune escape | | 7 |  |
| miR-9 | Nasopharyngeal cancer cell | Down | Promote angiogenesis of TME | | 8 |  |
| lncRNA *H19* | CAFs | Up | Participates in the glycolysis pathway of CAFs and promoting the progression of oral cancer | | 9 | |
| lncRNA *FLJ22447* | CAFs | Up | Promotes the transformation of CAFs | | 10 | |
| lncRNA *IFITM4P* | Oral cancer cell | Up | Promotes immune escape | | 11 | |
| lncRNA *DCST1-AS1* | Oral cancer cell | Up | Promote the polarization of M2 macrophages | | 12 | |
| circFAT1 | Oral cancer cell | Up | Promotes cancer stemness and immune evasion | | 13 | |

**Reference**

1 Li, Y. Y. *et al.* Cancer-associated fibroblasts contribute to oral cancer cells proliferation and metastasis via exosome-mediated paracrine miR-34a-5p. *EBioMedicine* **36**, 209-220, doi:10.1016/j.ebiom.2018.09.006 (2018).

2 Wang, X. *et al.* Loss of exosomal miR-3188 in cancer-associated fibroblasts contributes to HNC progression. *J Exp Clin Cancer Res* **38**, 151, doi:10.1186/s13046-019-1144-9 (2019).

3 Sun, L. P. *et al.* Cancerassociated fibroblastderived exosomal miR3825p promotes the migration and invasion of oral squamous cell carcinoma. *Oncol Rep* **42**, 1319-1328, doi:10.3892/or.2019.7255 (2019).

4 Qin, X. *et al.* Exosomal miR-196a derived from cancer-associated fibroblasts confers cisplatin resistance in head and neck cancer through targeting CDKN1B and ING5. *Genome Biol* **20**, 12, doi:10.1186/s13059-018-1604-0 (2019).

5 Zhu, G. *et al.* Small extracellular vesicles containing miR-192/215 mediate hypoxia-induced cancer-associated fibroblast development in head and neck squamous cell carcinoma. *Cancer Lett* **506**, 11-22, doi:10.1016/j.canlet.2021.01.006 (2021).

6 Amit, M. *et al.* Loss of p53 drives neuron reprogramming in head and neck cancer. *Nature* **578**, 449-454, doi:10.1038/s41586-020-1996-3 (2020).

7 Wu, X. *et al.* Down-regulation of the tumor suppressor miR-34a contributes to head and neck cancer by up-regulating the MET oncogene and modulating tumor immune evasion. *J Exp Clin Cancer Res* **40**, 70, doi:10.1186/s13046-021-01865-2 (2021).

8 Lu, J. *et al.* Exosomal miR-9 inhibits angiogenesis by targeting MDK and regulating PDK/AKT pathway in nasopharyngeal carcinoma. *J Exp Clin Cancer Res* **37**, 147, doi:10.1186/s13046-018-0814-3 (2018).

9 Yang, J. *et al.* Glycolysis reprogramming in cancer-associated fibroblasts promotes the growth of oral cancer through the lncRNA H19/miR-675-5p/PFKFB3 signaling pathway. *Int J Oral Sci* **13**, 12, doi:10.1038/s41368-021-00115-7 (2021).

10 Ding, L. *et al.* A novel stromal lncRNA signature reprograms fibroblasts to promote the growth of oral squamous cell carcinoma via LncRNA-CAF/interleukin-33. *Carcinogenesis* **39**, 397-406, doi:10.1093/carcin/bgy006 (2018).

11 Shi, L. *et al.* LncRNA IFITM4P promotes immune escape by up-regulating PD-L1 via dual mechanism in oral carcinogenesis. *Mol Ther* **30**, 1564-1577, doi:10.1016/j.ymthe.2022.01.003 (2022).

12 Ai, Y. *et al.* lncRNA DCST1-AS1 Facilitates Oral Squamous Cell Carcinoma by Promoting M2 Macrophage Polarization through Activating NF-kappaB Signaling. *J Immunol Res* **2021**, 5524231, doi:10.1155/2021/5524231 (2021).

13 Jia, L., Wang, Y. & Wang, C. Y. circFAT1 Promotes Cancer Stemness and Immune Evasion by Promoting STAT3 Activation. *Adv Sci (Weinh)* **8**, 2003376, doi:10.1002/advs.202003376 (2021).
