# Supplementary material for: Regulation of epigenetic modifications in the head and neck tumour microenvironment
Source: Front Immunol. 2022 Oct 28;13:1050982. doi: 10.3389/fimmu.2022.1050982 (PMC9667738; doi:10.3389/fimmu.2022.1050982)
Supplement: Supplementary file 3 [file Table_2.docx]

Table S2. Key proteins associated with epigenetic modifications in TME of head and neck tumours

| Key protein | Type | Epigenetic features | Expression level | Role | Ref |
| --- | --- | --- | --- | --- | --- |
| VEGF | Vascular endothelial growth factor | Hypomethylation | Up | Promote angiogenesis | 1 |
| FBLN2S | Tumour-suppressor  gene | Hypermethylation | Down | Promote angiogenesis | 2 |
| miR-30e-5p | ncRNA | - | Up | Inhibit angiogenesis and reduce tumor invasion and metastasis | 3 |
| OAT | - | Hypomethylation | - | Promote immune cell infiltration | 4 |
| SQLE | Oncogene | Demethylation | Up | Promote immune escape | 5 |
| DNMT1 | DNA methyltransferase | Maintaining DNA methylation | Up | Promote immunosuppressive microenvironment | 6 |
| TRMT61A | Writer | m^1^A modification was upregulated overall | Up | Negative correlation with immune checkpoints and CD8^+^T cells in TME | 7 |
| TRMT61B | Writer |  | Up |  |  |
| TRMT10C | Writer |  | Up |  |  |
| YTHDF1 | Reader |  | Up |  |  |
| YTHDF2 | Reader |  | Up |  |  |
| ALKBH1 | Eraser |  | Up |  |  |
| NOP2 | Writer | m^5^C modification was upregulated overall | Up | Decreased immune cell immune activity | 8 |
| NSUN3 | Writer |  | Up |  |  |
| NSUN4 | Writer |  | Up |  |  |
| NSUN5 | Writer |  | Up |  |  |
| NSUN6 | Writer |  | Up |  |  |
| TRDMT1 | Writer |  | Up |  |  |
| ALYREF | Reader |  | Up |  |  |
| YBX1 | Reader |  | Up |  |  |
| ALKBH1 | Eraser |  | Up |  |  |
| METTL3 | Writer | m^6^A modification | Down | Promote immunosuppressive | 9 |
| YTHDF1 | Reader |  | Up | Participate in TME immune regulation | 10 |
| IGF2BP2 | Reader |  | Up |  |  |
| YTHDC2 | Reader |  | Down | Related to the degree of TME immune infiltration | 11 |
| ALKBH5 | Eraser |  | Up | Promote immune escape | 12 |

**Reference**

1 Lu, Y. *et al.* Sevoflurane attenuate hypoxia-induced VEGF level in tongue squamous cell carcinoma cell by upregulating the DNA methylation states of the promoter region. *Biomed Pharmacother* **71**, 139-145, doi:10.1016/j.biopha.2015.02.032 (2015).

2 Law, E. W. *et al.* Anti-angiogenic and tumor-suppressive roles of candidate tumor-suppressor gene, Fibulin-2, in nasopharyngeal carcinoma. *Oncogene* **31**, 728-738, doi:10.1038/onc.2011.272 (2012).

3 Zhang, S. *et al.* miR-30e-5p represses angiogenesis and metastasis by directly targeting AEG-1 in squamous cell carcinoma of the head and neck. *Cancer Sci* **111**, 356-368, doi:10.1111/cas.14259 (2020).

4 Sun, Y. *et al.* Differential OAT methylation correlates with cell infiltration in tumor microenvironment and overall survival postradiotherapy in oral squamous cell carcinoma patient. *J Oral Pathol Med* **51**, 611-619, doi:10.1111/jop.13328 (2022).

5 Liu, Y., Fang, L. & Liu, W. High SQLE Expression and Gene Amplification Correlates with Poor Prognosis in Head and Neck Squamous Cell Carcinoma. *Cancer Manag Res* **13**, 4709-4723, doi:10.2147/CMAR.S305719 (2021).

6 Yang, S. C. *et al.* Inhibition of DNMT1 potentiates antitumor immunity in oral squamous cell carcinoma. *Int Immunopharmacol* **111**, 109113, doi:10.1016/j.intimp.2022.109113 (2022).

7 Gao, L. *et al.* The Impact of m1A Methylation Modification Patterns on Tumor Immune Microenvironment and Prognosis in Oral Squamous Cell Carcinoma. *Int J Mol Sci* **22**, doi:10.3390/ijms221910302 (2021).

8 Gao, L. *et al.* The RNA Methylation Modification 5-Methylcytosine Impacts Immunity Characteristics, Prognosis and Progression of Oral Squamous Cell Carcinoma by Bioinformatics Analysis. *Front Bioeng Biotechnol* **9**, 760724, doi:10.3389/fbioe.2021.760724 (2021).

9 He, J. *et al.* METTL3 restrains papillary thyroid cancer progression via m(6)A/c-Rel/IL-8-mediated neutrophil infiltration. *Mol Ther* **29**, 1821-1837, doi:10.1016/j.ymthe.2021.01.019 (2021).

10 Li, S., Wu, Q., Liu, J. & Zhong, Y. Identification of Two m6A Readers YTHDF1 and IGF2BP2 as Immune Biomarkers in Head and Neck Squamous Cell Carcinoma. *Front Genet* **13**, 903634, doi:10.3389/fgene.2022.903634 (2022).

11 Li, Y. *et al.* The m6A reader protein YTHDC2 is a potential biomarker and associated with immune infiltration in head and neck squamous cell carcinoma. *PeerJ* **8**, e10385, doi:10.7717/peerj.10385 (2020).

12 Jin, S. *et al.* The m6A demethylase ALKBH5 promotes tumor progression by inhibiting RIG-I expression and interferon alpha production through the IKKepsilon/TBK1/IRF3 pathway in head and neck squamous cell carcinoma. *Mol Cancer* **21**, 97, doi:10.1186/s12943-022-01572-2 (2022).
